# Effects of manufacturing errors of gear macro-geometry on gear performance

**DOI:** 10.1038/s41598-022-27204-9

**Published:** 2023-01-02

**Authors:** Woo-Jin Chung, Young-Jun Park, Chanho Choi, Su-Chul Kim

**Affiliations:** 1 Department of Biosystems Engineering, Seoul National University, 1 Gwanak-Ro, Gwanak-Gu, Seoul, 08826 South Korea; 2 Global Smart Farm Convergence, Seoul National University, 1 Gwanak-Ro, Gwanak-Gu, Seoul, 08826 South Korea; 3 Research Institute of Agriculture and Life Sciences, Seoul National University, 1 Gwanak-Ro, Gwanak-Gu, Seoul, 08826 South Korea; 4 Tractor Advanced Development Group, LS Mtron, Anyang, 14118 South Korea; 5 Department of Smart Industrial Machinery, Korea Institute of Machinery & Materials, Daejeon, 34103 South Korea

**Keywords:** Engineering, Mathematics and computing

## Abstract

In gear design, gear performance metrics such as safety factors for tooth root stress and surface durability, peak-to-peak static transmission error (PPSTE), efficiency, mass, and volume are considered. They are calculated by the geometric parameters of gear macro- and micro-geometry, and manufacturing errors related to gear geometry significantly affect gear performance. However, previous studies have only focused on the micro-geometry errors. In this study, Monte Carlo-type robustness analysis was performed considering the manufacturing errors for tooth thickness, tip diameter, and center distance. Gear performance metrics except PPSTE were calculated based on the international standards and geometric characteristics. PPSTE was evaluated analytically due to lack of standards. When the errors were considered, two gear pairs with the safety factors satisfying the design requirements and similar performance for PPSTE, efficiency, mass, and volume showed different gear performances. There were many samples in gear pair 1 that could not satisfy the design requirements of safety factors, and gear pair 2 had the robustness of PPSTE not only at the specific torque but also with wide torque range when compared to gear pair 1. These results imply that considering the gear macro-geometry errors and robustness of PPSTE is significantly important when designing gears.

## Introduction

Gears are a representative mechanical element for power transmission and widely used in various applications such as automobiles, aircraft, industrial machinery, construction machinery, and power plants. Lubrication is essential for the smooth use of gears, but they exhibit many advantages such as constant power transmission, high power density, and high efficiency. Depending on the application, minimum requirements related to durability and life, noise and vibration, power transmission efficiency, and weight are defined during design^1^.

Metrics for evaluating gear performance include safety factors for tooth root stress and surface durability, transmission error for gear vibration and noise, power transmission efficiency, volume, and mass. The safety factor is rated for the pinion and wheel, and the required minimum safety factors according to the application must be satisfied^2^. Gear vibration and noise are closely related to the peak-to-peak static transmission error (PPSTE), which is used as an evaluation metric for gear vibration and noise^3^. Efficiency of a gear pair is divided into load independent and load dependent power loss and is evaluated for not each gear but a gear pair. When designing gear specifications, only the load dependent power loss is usually considered^4,5^. Among the performance metrics of gears, the safety factors, efficiency, volume, and mass can be calculated based on the geometrical characteristics of the gear or several international standards. However, since the method of predicting PPSTE is not standardized, gear contact analysis must be conducted to evaluate PPSTE. The methods for predicting PPSTE are divided into finite element methods^6–9^, analytical-FEM hybrid methods^10–12^, and analytical methods^13–21^.

The formulas for gear rating include various factors, which consider the bending and contact stresses occurring in a gear pair as realistic values. Among them, only the coefficients considering the manufacturing accuracy are the internal dynamic factor, face load factor, and transverse load factor ^2^. The internal dynamic factor considers the internal dynamic effect caused by the gear tooth accuracy at the operating speed and applied load. As the effect of manufacturing accuracy on the internal dynamic factor has been confirmed by many previous studies for decades^22–25^, the calculation formula for it in ISO 6336 considers the manufacturing accuracy relatively accurately^26^. ISO 6336 presents the calculation method that considers manufacturing accuracy not only for the internal dynamic factor but also for the face load and transverse load factors. Although the proposed method cannot fully consider the manufacturing accuracy, it is relatively reliable^2^, and a gear designer could obtain those factors through a simple formula. However, to accurately consider manufacturing accuracy in the face load factor and transverse load factor, gear contact analysis is essential^26^.

For involute gears, gear performance metrics are determined by gear geometry, material, and operating conditions. The gear geometry could be divided into gear macro- and micro-geometry. The gear macro-geometry refers to basic geometric parameters such as normal module, normal pressure angle, helix angle, number of teeth, profile shift coefficient, and face width. Most of the parameters of the gear macro-geometry are determined by the shape and manufacturing method of the gear tooth cutting tool. The gear micro-geometry includes the tooth modification information such as amount and starting point of modification applied in the profile and lead direction of the gear flank.

Many studies on the robust design of gears have been conducted because manufacturing errors of micro-geometry have significant effects on the contact characteristics of a gear pair. Yu and ISHII^27^ presented a methodology to find the robust optimum considering manufacturing errors and various operating loads and applied it to gear design. They found that the optimal point without those considered may not be the robust optimal point. Harianto and Houser^28^ proposed a method for deriving the optimal gear micro-geometry to minimize noise and stress in a wide operating load range. In addition, by using the Monte-Carlo simulation method, a normal distribution was assumed for various micro-geometry errors, and 100 random samples were derived to perform a robust analysis. Houser^26^ analyzed the effect of the manufacturing errors of micro-geometry on the contact and root stress of gears and load distribution. It was confirmed that the factors for gear rating are significantly affected by micro-geometry errors in profile and lead and manufacturing variability of bias. Recently, the robust optimized design of the gear micro-geometry considering the ranges of torques and gear mesh misalignment was also performed^29^. Most previous studies considering the manufacturing accuracy were mainly conducted on the micro-geometry of the gear tooth, and the macro-geometry was assumed unaffected by the manufacturing errors of micro-geometry in the robust optimization problem.

However, in the design stage, the manufacturing accuracy of gears is usually defined for not only micro-geometry but also macro-geometry such as tooth thickness, tip diameter, and center distance. The manufacturing errors for these can change the gear performance. In addition, like the previous research on robust optimization problem for gear micro-geometry, considering only the manufacturing errors of micro-geometry in fixed macro-geometry, the gear performance metrics may not show a significant variance. From this point of view, it seems very valuable to evaluate how the gear performance metrics could be changed by considering manufacturing errors of macro-geometry.

The main novelty of this study is that the robustness analysis in which the same manufacturing errors of the macro-geometry were considered was performed for two gear pairs that have similar performance metrics when any manufacturing error was not considered. The performance metrics included safety factors for tooth root stress and surface durability, PPSTE, efficiency, volume, and mass. It was confirmed that it is necessary to select the gear macro-geometry in the design stage considering its manufacturing errors to improve the robustness of gear performance.

## Methods

### Gear performance metrics

#### Gear rating

In this study, the safety factors for tooth root stress and surface durability of pinion and wheel were calculated based on ISO 6336:2006 Method B^2^.

The safety factor for tooth root stress is a gear performance metric for evaluating the load capacity at which failure will not occur in the tooth root fillet during its design life. It is calculated using the maximum tensile stress at the tooth root surface and allowable bending stress. The calculation formula suggested in the standard is as follows:

1 $$\sigma_{F} = \frac{{F_{t} }}{{bm_{n} }}Y_{F} Y_{S} Y_{\beta } Y_{B} Y_{DT} K_{A} K_{V} K_{F\beta } K_{F\alpha }$$

2 $$\sigma_{FP} = \frac{{\sigma_{{F {\text{lim}}}} Y_{ST} Y_{NT} }}{{S_{{F {\text{min}}}} }}Y_{{\delta {\text{rel T}}}} Y_{{R{\text{ rel T}}}} Y_{X}$$

3 $$S_{F} = \frac{{\sigma_{FP} }}{{\sigma_{F} }}$$

where $\sigma_{F}$ denotes the tooth root stress; $F_{t}$ is the nominal tangential load, the transverse load tangential to the reference cylinder; $b$ and $m_{{\text{n}}}$ are face width and normal module of a gear; $Y_{F}$, $Y_{S}$, $Y_{{\beta }}$, $Y_{B}$, and $Y_{DT}$ denote form factor, stress correction factor, helix angle factor, rim thickness factor, and deep tooth factor, respectively; $K_{A}$ and $K_{V}$ represent application factor and internal dynamic factor; $K_{F\beta}$ is face load factor for tooth root stress; $K_{F\alpha}$ is transverse load factor for tooth root stress; ${\sigma }_{FP}$ denotes the permissible bending stress; $\sigma_{F{\text{ lim}}}$ is the allowable stress number for bending; $S_{F{\text{ min}}}$ represents the minimum required safety factor for tooth root stress; $Y_{ST}$, $Y_{NT}$, $Y_{{\delta {\text{ rel T}}}}$, $Y_{{R{\text{ rel T}}}}$, and $Y_{X}$ are stress correction factor from reference test gears, life factor for tooth root stress, relative notch sensitivity factor, relative surface factor, and size factor relevant to tooth root strength, respectively; $S_{F}$ represents the safety factor for tooth root stress.

The safety factor for surface durability is based on the Hertzian contact stress equation as an evaluation of the limit at which destructive fitting will not occur. It is calculated using the contact stress and allowable contact stress on the gear tooth surface. The calculation formula suggested in the standard is as follows:

4 $$\sigma_{H} = Z_{B, D} Z_{H} Z_{E} Z_{\varepsilon } Z_{\beta } \sqrt {\frac{{F_{t} }}{{d_{1} b}}\frac{u + 1}{u}K_{A} K_{V} K_{H\beta } K_{H\alpha } }$$

5 $$\sigma_{HP} = \frac{{\sigma_{{H {\text{lim}}}} Z_{NT} }}{{S_{{H{\text{ min}}}} }}Z_{L} Z_{V} Z_{R} Z_{W} Z_{X}$$

6 $$S_{H} = \frac{{\sigma_{HP} }}{{\sigma_{H} }}$$

where $\sigma_{H}$ denotes the contact stress; $Z_{B}$ and $Z_{D}$ are single pair tooth contact factors for the pinion and the wheel, respectively; $Z_{H}$, $Z_{E}$, $Z_{\varepsilon }$, and $Z_{\beta }$ represent zone factor, elasticity factor, contact ratio factor, and helix angle factor, respectively; $d_{1}$ denotes the reference diameter of pinion; $u$ is the gear ratio; $K_{H\beta}$ is face load factor for contact stress; $K_{H\alpha}$ is transverse load factor for contact stress; ${\sigma}_{HP}$ denotes the permissible contact stress; $\sigma_{H{\text{ lim}}}$ is the allowable stress number for contact; $S_{H{\text{ min}}}$ represents the minimum required safety factor for surface durability; $Z_{NT}$, $Z_{L}$, $Z_{V}$, $Z_{R}$, $Z_{W}$, and $Z_{X}$ are life factor for test gears for contact stress, lubricant factor, velocity factor, roughness factor, work hardening factor, and size factor for contact stress; $S_{H}$ represents the safety factor for surface durability.

Most of the factors required for gear rating were calculated based on ISO 6336:2006 Method B, but the face load factor and the transverse load factor were calculated based on Method C. However, some factors must be defined directly by the designer ^30^. Table 1 shows the factors defined for gear rating in this study. 18CrNiMo7-6 was selected as the material of pinion and wheel, which is case-hardened steel. Permissible stresses for tooth root stress and surface durability were defined based on the selected material. The lubricant viscosity was defined based on ISO VG 220. The minimum safety factors for tooth root stress and surface durability, usually determined by the designer according to the application, were selected as 1.4 and 1.1, respectively.

**Table 1:** Factors for gear rating (for pinion and wheel).

Description	Symbol	Value
Allowable stress number for bending (MPa)	${\upsigma }_{{{\text{Flim}}}}$	430
Allowable stress number for contact (MPa)	$\sigma_{{H{\text{lim}}}}$	1,500
Minimum required safety factor for tooth root stress	$S_{{F{\text{min}}}}$	1.40
Minimum required safety factor for surface durability	$S_{{H{\text{min}}}}$	1.10

#### Static transmission error calculation

When a gear pair has a perfect involute profile, completely rigid teeth, and no manufacturing errors, and is assembled in the correct position, there is no variation in its mesh stiffness during gear meshing. In other words, an ideal gear pair is in conjugate motion, and ratio of angular displacement is constant at all meshing positions. However, gear pairs actually exhibit transmission errors during gear meshing due to variations in this ratio induced by manufacturing and assembly errors, intentional microgeometry modification, and elastic deformation of teeth. In this study, only loaded static transmission error (LSTE) caused by elastic deformation of teeth with applied load was considered.

The gear contact analysis related to LSTE is a very complex nonlinear problem. Calculating the LSTE based on the finite element (FE) method is very accurate, but it has the limitation in that it requires a lot of computational and time-consuming cost because the FE model must be constructed with very small elements to consider manufacturing errors^19^. In other words, it is most reasonable to use the analytical method when the calculation is performed for numerous cases with various macro-geometry parameters in design stage. In this study, the LSTE was calculated using the analytical model of Chung et al.^21^, which predicts the transmission error by considering the exact tooth profile. LSTE is calculated as follows:

7 $${\text{LSTE}}_{i} = \frac{{F_{{{\text{norm}}}} \cos \beta_{b} }}{{{\text{TVMS}}_{i} }}$$

8 $${\text{TVMS}}_{i} = \mathop \sum \limits_{t = 1}^{{N_{t} }} \mathop \sum \limits_{j = 1}^{{N_{s} }} k_{i,j,t}$$

where ${\text{LSTE}}_{i}$ denotes the loaded static transmission error at the *i*th meshing position; $F_{{{\text{norm}}}}$ and ${\beta }_{b}$ represent normal force at the gear mesh and helix angle at the base circle, respectively; ${\text{TVMS}}_{i}$ is the time-varying mesh stiffness at the *i*th meshing position; ${N}_{t}$ and ${N}_{s}$ denote the number of slices and tooth pairs, respectively; ${k}_{{i},{j},{t}}$ represent the mesh stiffness at the *i*th meshing position of the *j*th sliced tooth in the *t*th contact pair.

The mesh stiffness of a gear pair consists of the load independent and load dependent stiffnesses. The former is the summation of the bending stiffness, shear stiffness, axial compressive stiffness, and foundation stiffness of tooth, while the latter is the contact stiffness of a gear pair, which is dependent on the applied force to contact surface. The meshing stiffness is calculated as follows, and each calculation is described in detail in the authors’ previous study^21^:

9 $$\frac{1}{{k_{i,j,t} }} = \frac{1}{{k_{ind}^{p} }} + \frac{1}{{k_{ind}^{w} }} + \frac{1}{{k_{h} \left[ {F_{t} } \right]}}$$

10 $$\frac{1}{{k_{ind}^{p,w} }} = \frac{1}{{k_{b}^{p,w} }} + \frac{1}{{k_{s}^{p,w} }} + \frac{1}{{k_{a}^{p,w} }} + \frac{1}{{k_{f}^{p,w} }}$$

11 $$\frac{1}{{k_{h} \left[ {F_{t} } \right]}} = \frac{2}{\pi b}\left[ {\left( {\frac{{1 - \nu_{p}^{2} }}{{E_{p} }}} \right)\left\{ {{\text{ln}}\frac{{2h_{xp} }}{{b_{con} }} - \frac{{\nu_{p} }}{{2\left( {1 - \nu_{p} } \right)}}} \right\} + \left( {\frac{{1 - \nu_{w}^{2} }}{{E_{w} }}} \right)\left\{ {{\text{ln}}\frac{{2h_{xw} }}{{b_{con} }} - \frac{{\nu_{w} }}{{2\left( {1 - \nu_{w} } \right)}}} \right\}} \right]$$

12 $$b_{{con}} = \left[ {{{\frac{{4F_{t} }}{{\pi b}}\left\{ {\left( {\frac{{1 - \nu _{p}^{2} }}{{E_{p} }}} \right) + \left( {\frac{{1 - \nu _{w}^{2} }}{{E_{w} }}} \right)} \right\}} \mathord{\left/ {\vphantom {{\frac{{4F_{t} }}{{\pi b}}\left\{ {\left( {\frac{{1 - \nu _{p}^{2} }}{{E_{p} }}} \right) + \left( {\frac{{1 - \nu _{w}^{2} }}{{E_{w} }}} \right)} \right\}} {\left( {\frac{1}{{r_{{cp}} }} + \frac{1}{{r_{{cw}} }}} \right)}}} \right. \kern-\nulldelimiterspace} {\left( {\frac{1}{{r_{{cp}} }} + \frac{1}{{r_{{cw}} }}} \right)}}} \right]^{{1/2}}$$

where ${k}_{{{{ind}}}}^{{{p}}}$ and ${{k}}_{{{{ind}}}}^{{{w}}}$ are the load independent stiffness of the pinion and wheel, respectively; $k_{h} \left[ {F_{{{t}}} } \right]$ is the contact stiffness dependent on applied force $F_{{{t}}}$; $k_{b}^{p,w}$, $k_{s}^{p,w}$, $k_{a}^{p,w}$, and $k_{f}^{p,w}$ represent the bending stiffness, shear stiffness, axial compressive stiffness, and foundation stiffness of a tooth of the pinion and wheel; $\nu_{p}$ and $\nu_{w}$ denote the Poisson’s ratio of the pinion and wheel, respectively; ${{E}}_{{{p}}}$ and ${{E}}_{{{w}}}$ are the Young’s modulus of the pinion and wheel, respectively; $h_{xp}$ and $h_{xw}$ represent the half tooth thickness of the pinion and wheel at a distance *x* away from the mesh point; ${{b}}_{{{{con}}}}$ denotes the half width of contact region on a tooth; ${{r}}_{{{{cp}}}}$ and ${{r}}_{{{{cw}}}}$ are the radius of curvature at the point of contact of the pinion and wheel, respectively.

LSTE varies during the gear mesh cycle. Therefore, PPSTE is defined as the difference between the maximum and minimum values of LSTE and calculated as follows:

13 $${\text{PPSTE}} = \mathop {\max }\limits_{{}} \left( {{\text{LSTE}}_{i} } \right) - \mathop {\min }\limits_{{}} \left( {{\text{LSTE}}_{i} } \right)$$

#### Efficiency calculation

To predict the power transmission efficiency of a gear pair, it is necessary to calculate the power loss during operation and amount of heat dissipated through the housing. Since this is a very complex process, ISO/TR 14179^4,5^ provides the method for this. The efficiency of a gear pair is divided into the load independent and load dependent power loss, and only the latter is usually considered when designing gears. In this study, the efficiency considering only the load dependent power loss of the gear pair was defined as the metric for the efficiency of the gear pair using ISO/TR 14,179–2^5^.

The load dependent power loss of the gear is calculated as follows:

14 $$P_{VZP} = P_{A} \mu_{mz} H_{V}$$

where ${{P}}_{{{{VZP}}}}$ represents the load dependent gear power loss; $P_{{{A}}}$ is the input power and calculated as $F_{{{t}}} v_{{{t}}}$; $v_{{\text{t}}}$ denotes the peripheral speed at the pitch circle; $\mu_{{{{mz}}}}$ and $H_{{{V}}}$ are as obtained by the following equations:

15 $$\mu_{{{{mz}}}} = 0.048\left( {\frac{{F_{bt} /b}}{{v_{\sum } \rho_{eq} }}} \right)^{0.2} \eta_{{{\text{oil}}}}^{ - 0.05} Ra^{0.25} X_{L}$$

16 $$F_{bt} = \frac{{F_{t} }}{{\cos \alpha_{t} }}$$

17 $$v_{\sum } = 2v_{t} \sin \alpha_{wt}$$

18 $$\rho_{eq} = \frac{{\rho_{c} }}{{\cos \beta_{b} }}$$

19 $$Ra = 0.5\left( {Ra_{1} + Ra_{2} } \right)$$

20 $$H_{V} = \frac{{\pi \left( {u + 1} \right)}}{{z_{1} u\cos \beta_{b} }}\left( {1 - \varepsilon_{\alpha } + \varepsilon_{1}^{2} + \varepsilon_{2}^{2} } \right)$$

where $\mu_{{{{mz}}}}$ is the average coefficient of friction; $F_{bt}$ denotes the nominal tooth normal force in the face section; $v_{\sum }$ represents the sum velocity; $\rho_{eq}$ is the equivalent radius of curvature; $\eta_{{{\text{oil}}}}$ denotes the dynamic viscosity of oil at the operating temperature; $Ra$ represents the arithmetic average roughness of the pinion and wheel; $X_{{{L}}}$ is the oil lubricant factor and defined by the type of lubricant; $\alpha_{wt}$ denotes the working pressure angle; $\rho_{{{c}}}$ represents the equivalent radius of curvature at the pitch point of contact; $Ra_{1}$ and $Ra_{2}$ are the roughness of the pinion and wheel, respectively; $z_{1}$ denotes the number of teeth of pinion; $\varepsilon_{\alpha }$ represents the transverse contact ratio; $\varepsilon_{1}$ and $\varepsilon_{2}$ are the addendum contact ratio of the pinion and wheel, respectively.

Consequently, the efficiency of a gear pair is calculated as follows:

21 $$\eta_{eff} = \frac{{P_{A} - P_{VZP} }}{{P_{A} }}$$

where ${\eta }_{{{{eff}}}}$ is the gear mesh efficiency.

#### Volume and mass calculation

The volume and mass of a gear pair are calculated based on the geometrical properties of gears. As this study focused on the manufacturing errors of gear macro-geometry, the blank shape of the gear such as the rim or web was not considered. In addition, the volume was defined as a metric of the minimum space occupied by the gearbox where the gear pair is mounted. The volume and mass are calculated as follows:

22 $$V = \left( {a_{w} + \frac{{d_{a1} }}{2} + \frac{{d_{a2} }}{2}} \right) \cdot \mathop {\max }\limits_{{}} \left( {d_{a1} , d_{a2} } \right) \cdot b$$

23 $$m = \frac{\pi b\rho }{4}\left\{ {\left( {d_{m1}^{2} - d_{i1}^{2} } \right) + \left( {d_{m2}^{2} - d_{i2}^{2} } \right)} \right\}$$

24 $$d_{m1,2} = \frac{{\left( {d_{a1,2} + d_{f1,2} } \right)}}{2}$$

where $V$ is the volume of gear pair; $a_{w}$ denotes the center distance; $d_{{{{a}}1}}$ and $d_{{{{a}}2}}$ represent the tip diameter of the pinion and wheel, respectively; $m$ is the mass of gear pair; $\rho$ denotes the gear density; $d_{{{{m}}1}}$ and $d_{{{{m}}2}}$ represent the mean diameter of the pinion and wheel, respectively; $d_{{{{i}}1}}$ and $d_{{{{i}}2}}$ are the inner diameter of the pinion and wheel; $d_{{{{f}}1}}$ and $d_{{{{f}}2}}$ denote the root diameter of the pinion and wheel.

### Manufacturing errors of gear macro-geometry

Although many previous studies have analyzed the effect of gear micro-geometry errors on gear contact characteristics and gear tooth root and contact stresses ^26–29^, research on macro-geometry errors rarely exists. The gear macro-geometry error is caused by the manufacturing accuracy for the tooth thickness, tip diameter, and center distance of the gear. According to Houser^26^, although these errors can affect gear performance, they have not been investigated and are sufficiently valuable for future studies. Therefore, in this study, the effect of manufacturing error of gear macro-geometry, not micro-geometry such as profile slope, profile curvature, lead slope, lead curvature, and bias, which were mainly dealt with in previous studies, on the gear performance metrics was confirmed.

Table 2 lists the macro-geometry information of two gear pairs used in this study. The performance metrics for these gear pairs not considering manufacturing errors of macro-geometry are shown in Table 3. The two gear pairs were selected as specifications showing similar performance in terms of mass, volume, and PPSTE while satisfying the minimum safety factors for tooth root stress and surface durability. The design variable space and design constraints related to the selection of these gear pairs were defined and presented by the manufacturer. Robustness analysis was performed by applying the manufacturing errors for tooth thickness, tip diameter, and center distance to each gear pair. The tolerances of each manufacturing error are listed in Table 4. The gear tooth thickness tolerance according to DIN 3967^31^ was cd25, which is usually used for standard mechanical engineering and heavy machinery including an agricultural tractor. Since the tip diameter tolerance to prevent the interference and improve lubrication is usually selected according to the experience of a manufacturer, the tolerance was considered as the general value used in the industrial machinery. The center distance tolerance according to ISO 286^32^ was js6, which is usually used. The same tolerances of each manufacturing error ​were applied to the pinion and wheel.

**Table 2:** Macro-geometry of gear pairs.

Description	Symbol	Gear pair 1	Gear pair 2
Normal module (mm)	$m_{n}$	2.5	3.0
Normal pressure angle (°)	$\alpha_{n}$	20	22.5
Helix angle (°)	$\beta$	5	15
Number of teeth of pinion	$z_{1}$	35	27
Number of teeth of wheel	$z_{2}$	47	36
Face width (mm)	b	20	20
Profile shift coefficient of pinion	$x_{n}^{*}$	− 0.451	0.370
Center distance (mm)	$a_{w}$	101	101

**Table 3:** Performance metrics of gear pairs without considering manufacturing errors.

Description	Symbol	Gear pair 1	Gear pair 2
Safety factor for tooth root stress of pinion	$S_{F1}$	1.001	1.680
Safety factor for tooth root stress of wheel	$S_{F2}$	1.153	1.669
Safety factor for surface durability of pinion	$S_{H1}$	1.068	1.216
Safety factor for surface durability of wheel	$S_{H2}$	1.095	1.257
Mass of gear pair (kg)	$m$	2.494	2.490
Volume of gear pair (mm^3^)	$V$	5.022E + 05	5.082E + 05
Efficiency of gear pair (%)	$\eta_{eff}$	99.205	99.441
PPSTE (μm)	–	3.137	3.137

**Table 4:** Manufacturing errors of gear macro-geometry.

Description	Minimum value	Maximum value
Tooth thickness tolerance (mm)	− 0.110	− 0.070
Tip diameter tolerance (mm)	− 0.100	0
Center distance tolerance (mm)	− 0.011	0.011

### Monte Carlo simulation

In general, the method of analyzing the effect of the manufacturing accuracy of the gear on the gear performance is to assume that the manufacturing error follows a normal distribution and use the Monte Carlo simulation ^26,28^. In this analysis, it is assumed that each manufacturing error variable has an independent normal distribution, which is randomly sampled from this distribution. The mean and standard deviation, which are components of a normal distribution, were calculated using the information on the manufacturing errors of gear macro-geometry shown in Table 4. The range of tolerances was assumed to be 6-sigma. Therefore, the formulas for the mean and standard deviation of each manufacturing error are as follows:

25 $$\mu = \frac{{\text{minimum value + maximum value}}}{2}$$

26 $$\sigma = \frac{{\text{maximum value - minimum value}}}{6}$$

where ${\upmu }$ denotes the mean of each manufacturing error; ${\upsigma }$ is the standard deviation of each manufacturing error. Using these two values, the normal distribution could be obtained as follows:

27 $$f_{X} \left( x \right) = \frac{1}{{\sqrt {2\pi } \sigma }}{\text{exp}}\left[ { - \frac{1}{2}\left( {\frac{x - \mu }{\sigma }} \right)^{2} } \right], - \infty < x < \infty$$

where ${\text{f}}_{{\text{X}}} \left( {\text{x}} \right)$ refers to the probability density function. As there were three types of the manufacturing errors, same number of normal distributions were obtained.

**Figure 1:**
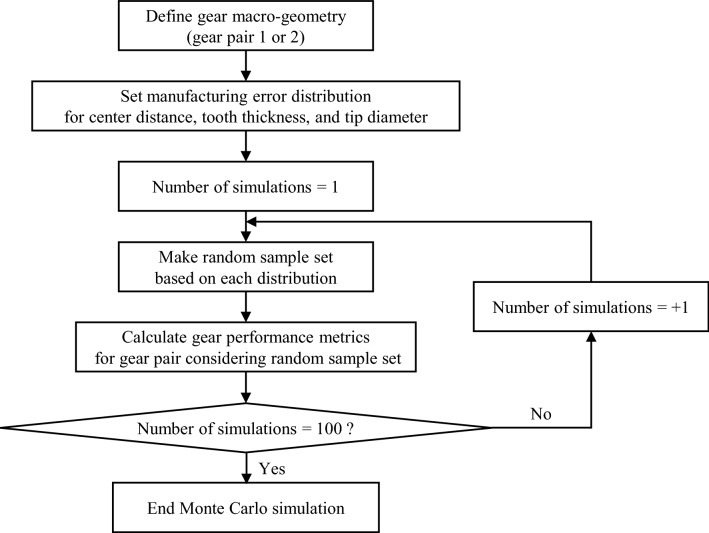
Flow chart of Monte Carlo simulation using 100 random sample sets for two gear pairs.

Figure 1 shows the flow chart of Monte Carlo simulation used in this study. Based on the normal distribution of each manufacturing error, 100 random sample sets for each gear pair (gear pair 1 and gear pair 2 in Table 2) were evaluated with the gear performance metrics. One random sample set consisted of values extracted as a random sample from the distribution of each manufacturing error.

## Results

**Figure 2:**
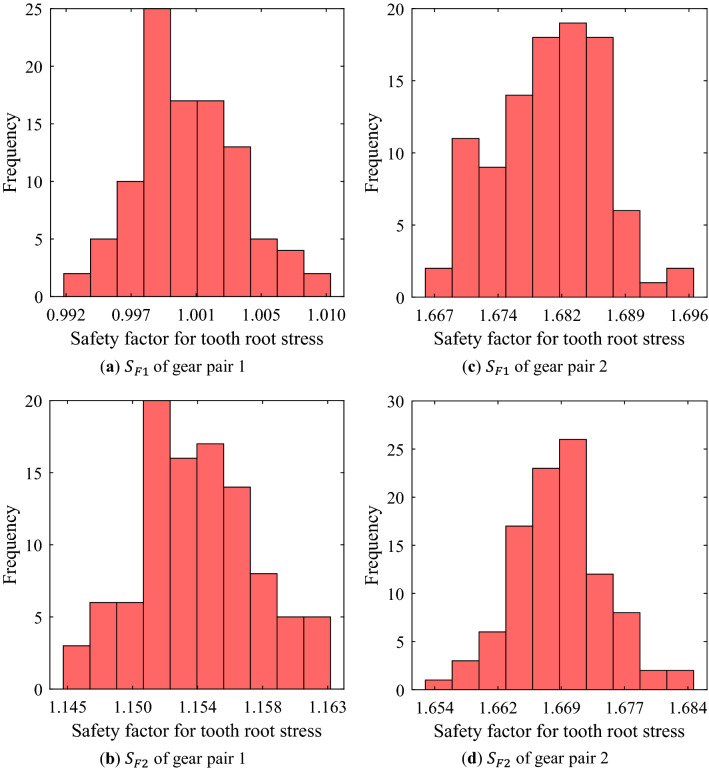
Frequency distribution of safety factor for tooth root stress of pinion and wheel of each gear pair using random robustness analysis.

**Figure 3:**
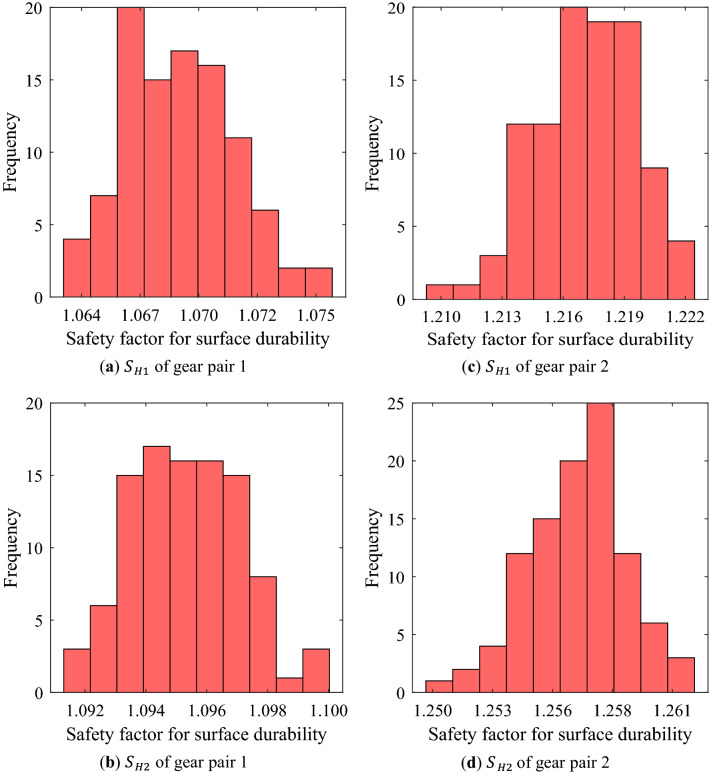
Frequency distribution of safety factor for surface durability of pinion and wheel of each gear pair using random robustness analysis.

**Figure 4:**
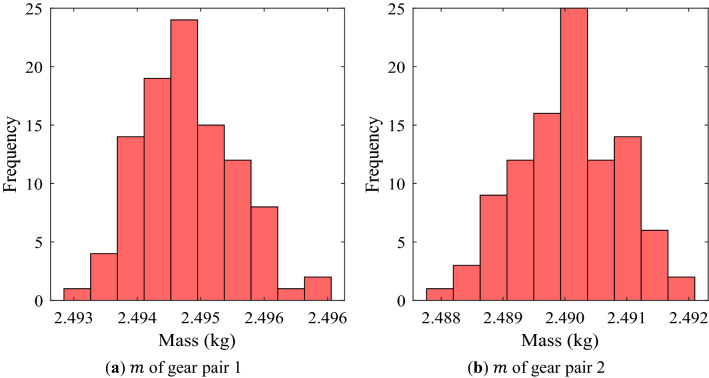
Frequency distribution of mass of each gear pair using random robustness analysis.

**Figure 5:**
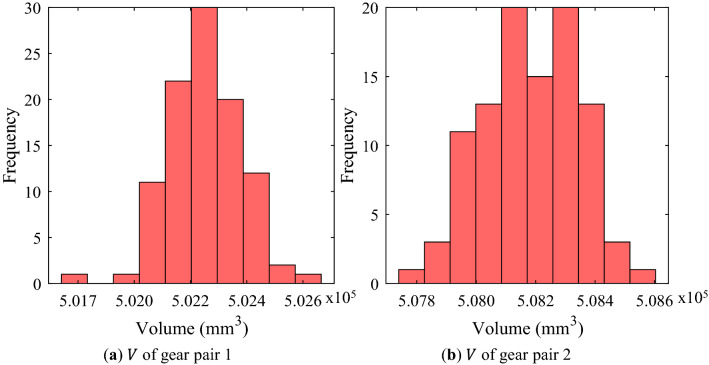
Frequency distribution of volume of each gear pair using random robustness analysis.

**Figure 6:**
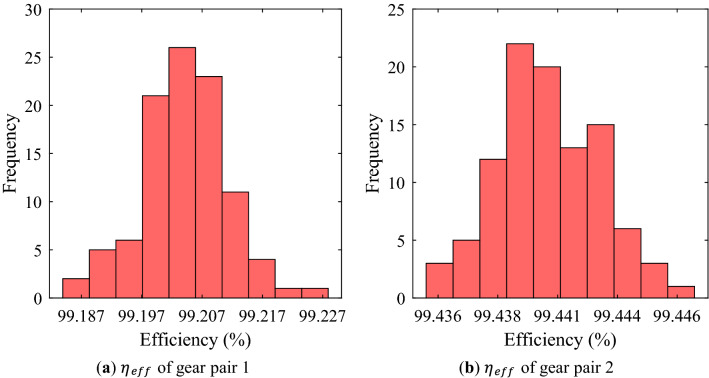
Frequency distribution of efficiency of each gear pair using random robustness analysis.

**Figure 7:**
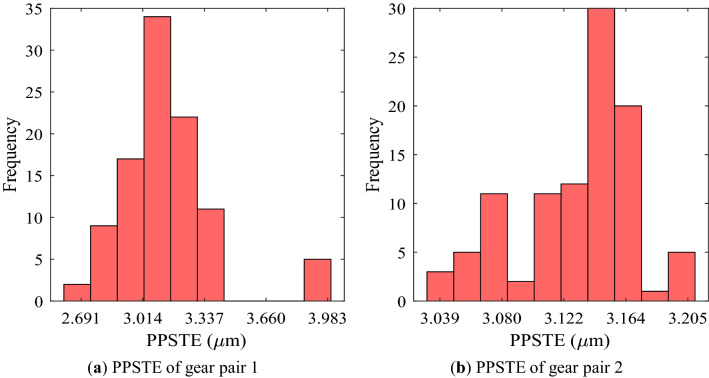
Frequency distribution of PPSTE of each gear pair using random robustness analysis.

Figures 2–7 shows the results of Monte Carlo-type robustness analysis for each gear performance metric considering manufacturing errors for tooth thickness, tip diameter, and center distance. For the analysis, the manufacturing errors listed in Table 2 were applied to all gears, pinions and wheels of gear pairs 1 and 2. The operating conditions are an input speed of 1383 rpm, an input torque of 300 Nm, and an operating time of 777 h, which are for the 4th gear pair in the main shift part of the transmission error of a 75-kW agricultural tractor^33^.

The distribution of the safety factors for tooth root stress and surface durability of gears according to the manufacturing errors of macro-geometry is not wide as shown in Figs. 2–3. However, the robustness analysis of the safety factor for tooth root stress of pinion of gear pair 1 (Fig. 2a) showed very significant results. When the gear macro-geometry errors were not considered, the safety factor was greater than 1.0, but there were many cases with the value less than 1.0 when various macro-geometry errors were considered.

From Figs. 4–5, it could be seen that the gear macro-geometry errors have little effect on the geometric performance metrics (mass and volume) of the gear pair. This is a result that could be easily predicted because the considered macro-geometry errors were not large enough to change the mass and volume. Similarly, it was confirmed that the efficiency of a gear pair was not significantly affected by the macro-geometry errors through Fig. 6.

Figure 7 shows the analysis results on PPSTE of each gear pair when the gear macro-geometry errors were considered. Although each error was assumed to be an independent normal distribution, the distribution of PPSTE for both gear pairs did not appear as a normal distribution, unlike other gear performance metrics. Moreover, the distribution of gear pair 2 was not large, whereas gear pair 1 had many samples that were somewhat far from the average value.

Table. 5 summarizes the results of the robustness analysis of the safety factors for tooth root stress and surface durability of each gear pair. Table. 6 summarizes the robustness analysis results of mass, volume, efficiency, and PPSTE of each gear pair. In Tables. 5 and 6, the normal values for each performance metric were calculated results (described in Table. 3) when the manufacturing errors of gear macro-geometry were not considered. Avg. and Stdv. mean the average and the standard deviation of the distribution for each performance metric when the errors were considered, respectively.

**Table 5:** Robust analysis results of safety factor for tooth root stress and surface durability of pinion and wheel of each gear pair.

Gear performance metrics	Normal value	Avg	Stdv	Avg.-3Stdv
${\text{S}}_{F1}$ of gear pair 1	1.001	1.000	0.004	0.988
${\text{S}}_{F1}$ of gear pair 2	1.680	1.680	0.006	1.662
${\text{S}}_{F2}$ of gear pair 1	1.153	1.154	0.004	1.142
${\text{S}}_{F2}$ of gear pair 2	1.669	1.669	0.005	1.653
${\text{S}}_{H1}$ of gear pair 1	1.068	1.069	0.004	1.057
${\text{S}}_{H1}$ of gear pair 2	1.216	1.217	0.002	1.210
${\text{S}}_{H2}$ of gear pair 1	1.095	1.095	0.002	1.089
${\text{S}}_{H2}$ of gear pair 2	1.257	1.257	0.002	1.250

**Table 6:** Robust analysis results of mass, volume, efficiency, and PPSTE of each gear pair.

Gear performance metrics	Normal value	Avg	Stdv	Avg. + 3Stdv
$m$ of gear pair 1 (kg)	2.494	2.494	0.001	2.497
$m$ of gear pair 2 (kg)	2.490	2.490	0.001	2.493
$V$ of gear pair 1 (mm^3^)	5.022E + 05	5.022E + 05	1.375E + 02	5.026E + 05
$V$ of gear pair 2 (mm^3^)	5.082E + 05	5.082E + 05	1.586E + 02	5.087E + 05
$\eta_{eff}$ of gear pair 1 (%)	99.205	99.204	0.007	99.226
$\eta_{eff}$ of gear pair 2 (%)	99.441	99.441	0.002	99.447
PPSTE of gear pair 1 (μm)	3.137	3.136	0.249	3.884
PPSTE of gear pair 2 (μm)	3.137	3.132	0.039	3.251

As a design requirement, the safety factor should be 1.0 or more. Therefore, the statistically significant value in the robustness analysis for the safety factor is Avg.-3Stdv. As mentioned above, in the case of the safety factor for tooth root stress of pinion of gear pair 1 whose normal value was close to 1.0, Avg. was 1.0, but Avg.-3Stdv. was calculated as 0.988. In the other gear pair, both Avg. and Avg.-3Stdv. were above 1.0.

Unlike the safety factor, the other gear performance metrics are required to be below a specific value in design process. Therefore, the statistically significant value in the robustness analysis results for them is Avg. + 3Stdv. There was no significant difference between the normal value and Avg. for most of the results. Moreover, in the cases of the mass, volume, and efficiency, the difference between Avg. and Avg. + 3Stdv. was not large because there were few samples with a value that deviated significantly from the normal value even if the manufacturing errors were considered. However, in the case of PPSTE, the Stdv. of gear pair 1 was about 6 times that of gear pair 2, and Avg. + 3Stdv. of gear pair 2 was 0.633 μm smaller than that of gear pair 1.

## Discussion

Among the basic geometric parameters of the gear macro-geometry, the profile shift coefficient and the center distance are mutually dependent. In the gear design process, when two values among the profile shift coefficients of pinion and wheel and center distance of a gear pair are defined, the other is automatically determined. The profile shift coefficient of pinion and the center distance are generally selected, and the profile shift coefficient of wheel is determined according to various considerations such as allowable stress, sliding velocity, and specific shape of the gear teeth.

When any manufacturing error is not considered, a gear pair has zero-backlash. However, when the tooth thickness tolerance is considered, normal backlash is included in the calculation, and the generating profile shift coefficient is calculated, not the profile shift coefficient. The center distance error is included in the calculation of the working transverse pressure angle, which is used to calculate the profile shift coefficient of the wheel, so it can be said that the center distance error indirectly affects the profile shift coefficient of the wheel. Unlike the tooth thickness and center distance errors, the tip diameter error affects not the basic geometric parameters of the gear macro-geometry but the contact characteristics of the gear. According to the change in the tip diameter, the contact characteristics related to gear meshing such ac start of active profile and end of active profile could be changed, which in turn causes a change in the tooth stiffness.

From the results of the robustness analysis, the safety factors for tooth root stress and surface durability, efficiency, mass, and volume showed a normal distribution when the manufacturing errors for the macro-geometry of each gear pair were assumed to be independent normal distributions. For these gear performance metrics, there was no significant difference between the normal value without the manufacturing errors considered and Avg. In addition, Stdv. was relatively smaller. In other words, even when the manufacturing errors were considered, the distribution of the gear performance metrics corresponding to the 6-sigma was not wide. However, when defining a value very close to the design requirement, such as the safety factor for tooth root stress of the pinion of gear pair 1 in this study, a large number of samples did not satisfy the design requirement when the manufacturing errors of gear macro-geometry were considered. These results show that even though the manufacturing errors for the macro-geometry has a small effect on the gear performance metrics, it is very important that the robust design considering the manufacturing errors of gear macro-geometry is essential for all manufactured gears to satisfy the design requirements.

Among the gear performance metrics, the frequency distribution of PPSTE was difficult to consider as a distinctly normal distribution in the robustness analysis. In particular, from the results of gear pair 1, there were many cases where PPSTE did not appear at some specific values but showed a large difference from Avg. As the contact characteristics of gears are very complex non-linear problems, it seems that they change non-linearly according to the gear macro-geometry and the manufacturing errors. The Stdv. of gear pair 1 was about 6 times that of gear pair 2. Accordingly, the Avg. + 3Stdv. of gear pair 2 was 0.633 μm smaller than that of gear pair 1. This was a very significant difference in value; therefore, it is essential to perform a robustness analysis considering the manufacturing errors of gear macro-geometry for PPSTE among gear performance metrics so that it is important to prevent unexpected cases where the design requirements are not satisfied.

**Figure 8:**
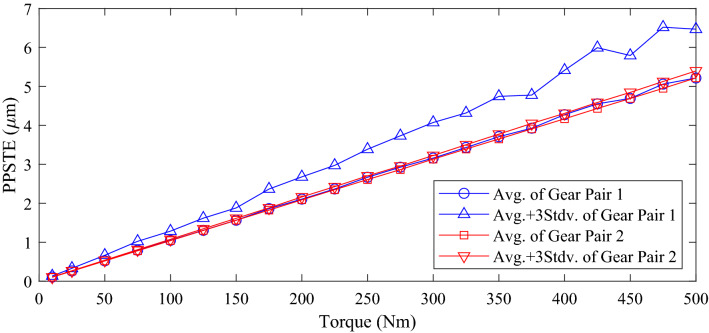
Robustness analysis results (Avg. and Avg. + 3Stdv.) for PPSTE according to torque.

Through the results of the robustness analysis of the gear performance metrics, it was confirmed the manufacturing errors of gear macro-geometry must be considered when designing gears. In particular, during the macro-geometry optimization, constraints and objective functions are set based on the performance metrics of each gear. Considering the influence of manufacturing errors, it is expected that the derived optimal solution could be changed. For example, in the optimization problem considering macro-geometry errors, the robustness could be ensured by setting Avg.-3Stdv. as constraints of the safety factors for tooth root stress and surface durability. In addition, by defining the objective function to Avg. + 3Stdv. of PPSTE instead of the normal value, it is possible to obtain the robust optimum. When diverse loads or large load variations are applied to a vehicle, the macro-geometry optimization is more suitable than the micro-specification optimization ^31^. For the robust optimum, the robustness of the PPSTE according to the torque could also be investigated as shown in Fig. 8. As the applied torque increases, although the difference between the normal value and Avg. + 3Stdv. of gear pair 1 ​​gradually widens, it was confirmed that gear pair 2 was robust against the manufacturing errors of gear macro-geometry under all torque conditions. Therefore, it seems essential to secure the robustness of the PPSTE by considering the manufacturing errors of gear macro-geometry when performing macro-geometry optimization under various load conditions.

## Conclusions

In this study, Monte-Carlo type robustness analysis of gear performance metrics was performed considering the manufacturing errors for tooth thickness, tip diameter, and center distance of gears. Safety factors for tooth root stress and surface durability, PPSTE, efficiency, mass, and volume were considered as the gear performance metrics. The safety factors and efficiency were calculated based on the international standards, and the mass and volume were calculated based on geometric characteristics of the gear pairs. The PPSTE was predicted based on a previous study by the authors because there is no standardized method.

The two gear pairs used in the robustness analysis satisfied the design requirements of the minimum safety factors for tooth root stress and surface durability when the manufacturing errors of gear macro-geometry were not considered. They also had similar performance in terms of mass, volume, and PPSTE. However, when the manufacturing errors of gear macro-geometry were taken into consideration, there were a number of samples that did not satisfy the design requirement of the safety factor for tooth root stress of the pinion of gear pair 1. In addition, PPSTE related to the contact characteristics of gears was confirmed to have a large nonlinearity due to the manufacturing errors of gear macro-geometry, and the PPSTE of gear pair 2 was much more robust than that of gear pair 1.

Through the results of robustness analysis of various gear performance metrics, this study provides the following original contributions: firstly, two gear pairs that have similar performance metrics when the manufacturing errors related to the macro-geometry are not considered show different robustness when those errors are considered; secondly, it seems essential to consider Avg.-3Stdv., not the normal value, when selecting gear macro-geometry parameters that barely satisfy the design requirements of the safety factors for tooth root stress and surface durability to prevent gear failure; finally, robustness must be ensured by selecting gear macro-geometry parameters based on Avg. + 3Stdv. rather than the normal value for PPSTE when the robustness of noise and vibration according to torques is considered as important factors for the designed gearbox system. However, in this study, it was not investigated how much the manufacturing errors of gear macro-geometry influenced on the macro-geometry optimization. In the future, optimization will be performed by setting the gear performance metrics considering the manufacturing errors for tooth thickness, tip diameter, and center distance as constraint and objective functions. In addition, the interactive effects of the manufacturing errors of gear macro- and micro- geometry on the gear performance metrics would be investigated by considering both errors.

## Data Availability

The datasets generated during and/or analyzed during the current study are available from the corresponding author on reasonable request.
